# A commercially available DJ-1 antibody does not detect *C. elegans* DJR-1.1 or DJR-1.2 proteins in whole worm lysates

**DOI:** 10.17912/micropub.biology.002375

**Published:** 2026-09-15

**Authors:** Hailey X. Egido-Betancourt, Cheyenne E. Brown, Farr Niere, Jyoti Iyer

**Affiliations:** 1 Department of Biology, North Carolina A&T State University, Greensboro, NC, United States

## Abstract

Custom antibody synthesis is both expensive and time-consuming. Therefore, the ability to repurpose existing commercially available antibodies to detect their corresponding *C. elegans* orthologs can be very beneficial. Here, we attempted to use the commercially available DJ-1 antibody NB300-270 to detect *C. elegans* DJR-1.1 and DJR-1.2 proteins in whole worm lysates. Our results indicate that the NB300-270 DJ-1 antibody does not detect *C. elegans* DJR-1.1 or DJR-1.2 proteins in whole worm lysates *.*

**Figure 1. Novus Biologicals NB300-270 DJ-1 antibody does not detect C. elegans DJR-1.1 or DJR-1.2:** Top panel) Western blot showing non-specific bands detected by the anti-DJ-1 antibody NB300-270 in *C. elegans.* Whole worm lysates from *djr-1.2 ( tm1346 )* , *djr-1.2 ( tm951 ), and djr-1.1 ( tm918 )* exhibit bands identical to those observed in wild-type ( N2 ) whole worm lysates. Bottom panel) Housekeeping protein α-tubulin demonstrates equal loading of samples. The image shown is representative of three independent experiments.

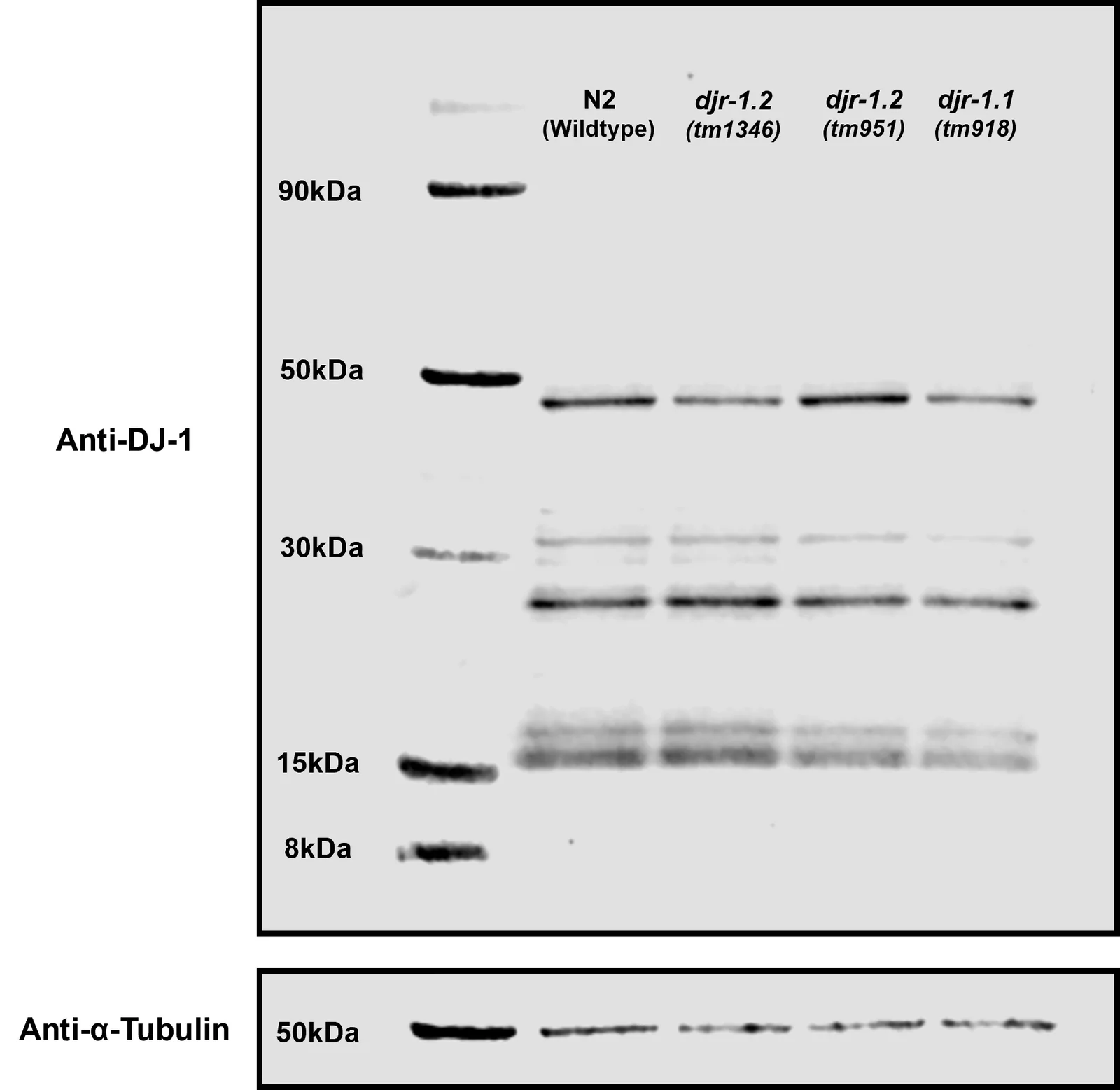



## Description

*Caenorhabditis elegans* ( *C. elegans* ), is a self-fertilizing nematode, that has been established as a model organism for neurodegenerative disease because the use of mammalian models, while invaluable, are subject to ethical considerations which make them expensive and time consuming (Chakraborty et al., 2013; Harrington et al., 2010). Antibodies are essential tools necessary for investigating biological research and while the vast majority of commercially available antibodies target mammalian proteins, few such reagents are available to detect *C. elegans* proteins (Hadwiger et al., 2010). One such protein, is Parkinson's disease (PD)-associated gene 7 (PARK7/DJ-1). DJ-1 protein is ubiquitously expressed in most mammalian tissues, primarily the brain within the cytosol and mitochondria (Chakraborty et al., 2013). In *C. elegans* , DJ-1 exists as two distinct orthologs of the human DJ-1 ( *PARK7* ) gene, *djr-1.1* and *djr-1.2* . DJR-1.1 primarily localizes to the intestinal cells while DJR-1.2 is expressed in neurons (Chakraborty et al., 2013; Lee et al., 2012).

In classic mammalian physiology, mutations in *PARK7* gene alter the structure and function of the encoded protein DJ-1, which increases the risk of developing autosomal recessive PD (Cooper & Van Raamsdonk, 2018; Skou et al., 2024). DJ-1 belongs to a large and functionally diverse homologous family, primarily implicated in oxidative stress by detoxing the cells' excess accumulation of glyoxal and methylglyoxal stress factors (Lee et al., 2012, 2013). Recently, DJ-1 has been shown to have RNA-binding activity (Niere et al., 2023; van der Brug et al., 2008). Indeed, we have demonstrated that DJ-1 binds to the mRNA coding for the alpha and auxiliary Ca ^2+^ channel subunits Ca _V_ 1.2 and α2δ2, and represses their mRNA translation in preclinical models of tuberous sclerosis complex (TSC) and Alzheimer's disease (AD) that exhibit overactive mammalian/mechanistic target of rapamycin (mTOR) or mTORopathy (Niere et al., 2023). As calcium is widely held as the most common second messenger, repression of these channels can profoundly alter calcium-dependent cellular processes such membrane excitability, transcription and translation (Kennedy, 1989). Since mammalian DJ-1 shares a conserved structure and function with *C. elegans* DJR-1.1 and DJR-1.2 , *C. elegans* could serve as a useful system to investigate other mTOR/DJ-1-dependent pathways ( *djr-1.1 (Gene) - WormBase : Nematode Information Resource* , 2026; *djr-1.2 (Gene) - WormBase : Nematode Information Resource* , 2026). Therefore, to investigate the roles of the *C. elegans* DJR-1.1 and DJR-1.2 proteins, we tested whether the commercially available Novus Biologicals antibody NB300-270, which has been reported to detect DJ-1 in several species, including human, mouse , rat, bovine, golden Syrian hamster, and zebrafish, can detect *C. elegans* DJR-1.1 or DJR-1.2 in whole worm lysates.

To evaluate the specificity of the bands detected by the Novus NB300-270 antibody in *C. elegans* whole worm lysates, we utilized the following mutant alleles: *djr-1.2 ( tm1346 )* , *djr-1.1 ( tm918 )* and *djr-1.2 ( tm951 ).* The *djr-1.1 ( tm918 )* strain contains an 825-bp deletion that removes the first two annotated exons of *djr-1.1* , whereas *djr-1.2 ( tm1346 )* contains a complex 1,035-bp deletion accompanied by a 378-bp insertion. Both *djr-1.1 ( tm918 )* and *djr-1.2 ( tm1346 )* are predicted null alleles (Cornejo Castro et al., 2010). In contrast, *djr-1.2 ( tm951 )* contains a 729-bp deletion located upstream of the annotated *djr-1.2* gene, which may result in an altered expression of *djr-1.2* through disruption of regulatory sequences (The *C. elegans* Deletion Mutant Consortium, 2012) ( **Reagents** ). Using western blotting, we demonstrate that the Novus Biologicals NB300-270 DJ-1 antibody is unable to detect either DJR-1.1 or DJR-1.2 in *C. elegans* whole-worm lysates, as all lanes display similar bands that are not altered in the mutant strains ( **Figure 1, top panel** ). Furthermore, the antibody fails to detect bands at the expected molecular weight of DJR-1.1 or DJR-1.2 (~20 kDa), indicating that the observed bands are non-specific. Importantly, the blot for the loading control (α-tubulin) shows that similar amounts of whole worm lysates were loaded for all samples ( **Figure 1, bottom panel** ). These data collectively indicate that the bands detected by the DJ-1 antibody are non-specific and that this antibody cannot be used to reliably detect *C. elegans* DJR-1.1 or DJR-1.2 in whole worm lysates.

## Methods

***C. elegans* *growth and maintenance***

All *C. elegans* strains were grown on MYOB agar plates (3.49 mM Tris-Cl, 1.98 mM Tris-base, 0.31% (w/v) bactopeptone, 34.2 mM sodium chloride, 8 μg/ml cholesterol, 2% (w/v) agar) seeded with OP50 bacteria and maintained at 20°C (Ashraf et al., 2026). The strains used in this study are listed in **Reagents** .

***Preparation of C. elegans lysates for immunoblotting***

*C. elegans* lysates for immunoblotting were adapted from Xie, S. et al. 2022 and prepared as follows. Briefly, 100 gravid adults for each genotype were picked using a platinum pick into 1ml of M9 buffer (3g KH _2_ PO _4_ , 6g Na _2_ HPO _4_ , 5g NaCl, 1ml 1M MgSO _4_ dissolved in 1 liter of H _2_ O). The worms were washed two times in 1ml of M9 buffer by centrifuging them at 300 x ***g*** for 5min. The supernatant was discarded and the worm pellet was then resuspended in 35µl of Laemmli SDS sample buffer, reducing (4X) (Thermo Fisher Scientific, MA, USA), heated at 95°C for 10 min and stored at −30°C until further use (Xie et al., 2022).

***Immunoblotting of C. elegans lysates***

*C. elegans* lysates were subjected to reducing SDS-PAGE electrophoresis followed by the dry blotting method. Briefly, 12µl of each worm lysate was loaded onto each well of a 4-20% SurePAGE™ Precast Bis-Tris Gel (Genscript Inc.) ( **Reagents** ). The gels were run at a constant current of 20mA for 15min then switched to 30mA for 1hr until sufficient band separation was achieved. The proteins were transferred from the gel onto a 0.2µm PVDF membrane using the Invitrogen iBLOT2 dry transfer method using the following 7min protocol (20V for 1min; 23V for 4min; 25V for 2min). The membranes were blocked using a blocking buffer (1× Tris-buffered saline, 0.5% Fish Gelatin, 0.1% Tween 20, 0.02% Sodium Azide) then incubated with rabbit anti-DJ-1 antibody (1:1000; Novus Biologicals, NB300-270) overnight at 4°C or a mouse anti-alpha tubulin antibody (1:200; Santa Cruz Biotechnology, SC-32293) at room temperature for several hours. The membrane was washed three times with 1× Tris-buffered saline, (19.8mM Tris-base, 150 mM NaCl; TBS) and incubated with the goat anti-rabbit-IgG 800CW secondary antibody (1:4000, LI-COR Biosciences, Inc.) for 4hrs, or with the goat anti-mouse-IgG 680RD secondary antibody (1:10,000; LI-COR Biosciences, Inc.) for 2hrs, washed three times with TBS, and imaged using the LI-COR Odyssey CLx imager (LI-COR Biosciences, Inc.) ( **Reagents** ).

## Reagents

**Table:** 

STRAIN/ REAGENT NAME	GENOTYPE/ HOST	CATALOG NUMBER	RESOURCE/ COMPANY
N2	wild-type	n/a	CGC
*djr-1.2 ( tm1346 ) *	*djr-1.2 ( tm1346 ) V *	n/a	NBRP
*djr-1.2 ( tm951 ) *	*djr-1.2 ( tm951 ) V *	n/a	NBRP
*djr-1.1 ( tm918 ) *	*djr-1.1 ( tm918 ) II *	n/a	NBRP
Park7/DJ-1 Antibody (Polyclonal)	Rabbit	NB300-270	Novus Biologicals
α-Tubulin (DM1α)	Mouse	SC-32293	Santa Cruz Biotechnology
IRDYE ® 800CW Goat Anti-Rabbit-IgG Secondary Antibody	Goat	926-32211	LI-COR Biosciences
IRDYE ® 680RD Goat Anti-Mouse-IgG Secondary Antibody	Goat	926-68070	LI-COR Biosciences
iBlotTM 2 PVDF Mini Stacks	n/a	IB24002	Invitrogen
Laemmli SDS sample buffer, reducing (4X)	n/a	J60015.AD	ThermoScientific
SurePAGETM, Bis-Tris, 10x8 wells	n/a	M00656	GenScript
Chameleon ® Kit 700 Pre-stained Protein Ladder	n/a	928-90000	LI-COR Biosciences
Chameleon ® Kit 800 Pre-stained Protein Ladder	n/a	928-90000	LI-COR Biosciences
Tris Base	n/a	BP152-500	Fisher Bioreagents
Sodium Chloride	n/a	S3014-500G	Sigma Life Science
Tris hydrochloride	n/a	10812846001	Roche
BactoTM Peptone	n/a	211677	Gibco
Cholesterol	n/a	C8667-25G	Sigma Life Sciences
Select Agar	n/a	30391-049	Invitrogen
Magnesium sulfate heptahydrate	n/a	M2773-500G	Sigma Life Sciences
Potassium Phosphate Monobasic	n/a	BP362-500	Fisher Bioreagents
Gelatin from cold water fish skin	n/a	G7041-500G	Sigma Aldrich
Sodium phosphate, dibasic	n/a	34242-4	Aldrich Chemical Company Inc.
Tween ® 20, ultra pure	n/a	J20605	ThermoScientific
Sodium Azide	n/a	S24080-250.0	Research Products International
